# Connecting Future Environmental Trends and Assessments of Fish and Wildlife Resources of Concern: A Case Study of Big Pine Key, Florida

**DOI:** 10.3390/su142114553

**Published:** 2022-11-05

**Authors:** Lori A. Miller, Matthew C. Harwell

**Affiliations:** 1 Florida, Caribbean, and Gulf Coast Complex National Wildlife Refuges, U.S. Fish and Wildlife Service, 1339 20th Street, Vero Beach, FL 32960, USA; 2 Pacific Ecological Systems Division, U.S. Environmental Protection Agency, 2111 SE Marine Science Drive, Newport, OR 97365, USA

**Keywords:** climate change, adaptation strategies, stakeholder engagement, sea-level rise, fish and wildlife

## Abstract

Changes in hydrologic and climatic trends will influence the ecology of Florida, and climate scenarios agree that many areas of Florida are susceptible to sea-level rise impacts. The U.S. Fish and Wildlife Service’s Climate Change Action Program focuses on a framework to examine climate change effects on fish, wildlife, plants, and habitats of all three. To follow the program, this study examines how to incorporate current scientific knowledge about regional climate projections in U.S. Fish and Wildlife Service analyses. It provides climate change and sea-level rise projections based on 2017 projections, information on changes in tropical cyclones, temperatures, and precipitation. This study also examines future effects of sea-level rise on existing habitat from saltwater intrusion of the freshwater lens below Big Pine Key. Projections of future sea-water elevations will periodically be reached or exceeded well before 2040 from short-term, stochastic, and extreme events (e.g., king tides and storm surge), and will increasingly inundate the root zone before complete saltwater intrusion. Future trends were connected to 2017 stakeholder-driven conversations about adaptation strategies to develop a suite of actions for creating temporary or permanent freshwater resources. However, beyond 3 ft (0.9 m) of sea-level rise, there are few adaptation options available for the Florida Key deer beyond relocations outside of the Florida Keys. Overall, the approach of connecting future environmental trends to assessments of fish and wildlife resources of concern can be transferred to other situations. Additionally, this approach can be used to update these analyses, such as with the recent 2022 sea-level rise updates by the National Oceanic and Atmospheric Administration, released after this work was conducted.

## 1. Introduction

Examining how climate change affects ecosystems involves integration and analysis of multiple, interdisciplinary data streams. These include cataloging existing environmental and ecological condition data, the use of climate scenario models, and the consideration of additional, co-occurring stressors. Translating these information sources to understanding resultant ecological effects of climate change impacts is a precursor to develop conservation, mitigation, adaptation, and/or resilience-focused ecosystem based management plans. Most importantly, translating climate and ecological effects to decision makers in an understandable fashion takes creativity and sometimes simpler approaches.

In the United States, the U.S. Fish and Wildlife Service (USFWS) is responsible for overseeing the Endangered Species Act (16 U.S.C. § 1531 *et seq*.) and a number of other regulations that protect fish, wildlife, plants, and habitats of all three. The USFWS Climate Change Action Program (CCAP) focuses on a broad framework to examine how climate change is affecting fish, wildlife, plants, and habitats of all three [1]. The CCAP implements sustainable climate change practices by requiring the use of best available climate science and the development and implementation of adaptation strategies at multiple landscape scales. Primary goals of the 2010 USFWS Climate Change Strategic Plan were: the sharing of scientific capacity within the conservation community; application of principles of Strategic Habitat Conservation [2]; and leading the conservation community in developing a “shared vision” for addressing climate change. Additionally, the literature contains examples for consideration of climate change in the endangered species context [3], Habitat Conservation Plans [4], recovery plans [5], and the National Wildlife Refuge system in general [6].

Scenario planning is a comprehensive exercise that involves development of scenarios that capture a range of plausible future conditions [7] that allows decision makers to explore multiple options and see if a management action is common across the majority of scenarios, potentially indicating greater support for that action [7,8]. Under changing climate conditions, the use of scenario planning becomes of paramount importance for evaluating resource lands, species, and habitats of concern. Whereas predictions and forecasts are statements about what will happen in the future with some degree of certainty, scenarios represent plausible, alternative characterizations of the future not intended to be associated with probabilities. Scenarios can be constructed as qualitative narrative storylines or quantitative expressions of future environmental conditions, depending on the outcomes needed to achieve the goal of a given planning effort [7]. That scenario development is then followed by an assessment of potential effects of those scenarios on a focal resource or decision, and identification of responses under each scenario, with a focus on those that are robust across scenarios.

This study examines how to approach connecting future environmental trends and assessments of fish and wildlife resources of concern with an emphasis on integrating climate change information into analyses and inform adaptation strategies. Using a case study of the middle Florida Keys, this paper examines how to incorporate best available science about regional climate change projections (scenarios) into decisions and planning activities along with ideas for presenting this science to decision makers in a more understandable way. As much as 30% of the habitat in coastal National Wildlife Refuges in the U.S. Southeast is expected to be flooded as a result of rising seas. In the Florida Keys, which experienced a rise in sea level of 23 cm between 1913 and 2006, 90% of land is 1.5 m or less above sea level [9]. Projecting the future condition of a species and future environmental condition, including sea-level rise (SLR), is integral to ecosystem-based management (EBM) adaptation planning. In this study, we connected findings to outcomes of stakeholder-driven workshops focused on identifying impacts to species under different SLR scenarios to inform adaptation planning, including prioritization of actions, examining risks, and examining adaptive capacity and governance issues, including describing species-specific actions.

### Study Site

The National Key Deer National Wildlife Refuge (Refuge), comprised of nearly 9000 acres (approximately 3600 ha) of land, was established in 1957 (16 U.S.C. 696-696b; Public Law 85–164 (71 Stat. 412)) to protect and conserve Florida Key deer (*Odocoileus virginianus clavium*), habitat for hundreds of endemic and migratory species, 21 Federally listed species, and a variety of plants endemic to the Florida Keys. The 2009 15-year Comprehensive Conservation Plan (CCP) for the Lower Florida Keys National Wildlife Refuge Complex [10] includes a focus on a proactive and adaptive ecosystem-management approach to promote “natural diversity and abundance of habitats for native plants and animals, especially Florida Keys’ endemic, trust, and keystone imperiled species” [10]. Efforts identified in the CCP include: protecting environmentally sensitive habitat; providing sanctuary for nesting and migratory birds; enhancing biological diversity and resiliency of fire-dependent pine rocklands; and enhancing fire-adapted habitat features that benefit priority species in the Refuge. The CCP management strategies include focus on pine rockland, salt marsh transition, freshwater wetland habitats, and island beach berm communities while looking towards understanding effects of natural events and global climate change, including SLR.

## 2. Materials and Methods

This study involved compiling secondary data and analyzing information among four elements: (1) inventory of existing conditions; (2) examination of SLR scenarios leading to evaluation of habitat impacts; (3) characterization of additional stressors; and (4) use of stakeholder workshops to translate information for adaptation action planning.

### 2.1. Inventory of Existing Conditions

Information on existing conditions in the middle Florida Keys were compiled, including information on: the topography of Big Pine Key; the freshwater lens below the islands, and resulting ground surface inundation and root zone salinization; SLR to date and resulting historical impacts; and information about threatened and endangered species.

### 2.2. Sea-Level Rise Scenarios and Resultant Habitat Impacts

The U.S. Geological Survey (USGS) in the National Oceanic and Atmospheric Administration (NOAA) 2020 updated report recommends the inclusion of impacts from tidal flooding and increasing high tides (an additional 0.30 m) in the Florida Keys. These are considered a high-risk factor for habitat and freshwater pools on the Florida Keys. To address this need, regional SLR projections were updated [11,12] and examined here. Revisions included recent observational and modeling literature related to the potential for rapid ice melt in Greenland and Antarctica along with other regional processes used to provide climate information at smaller scales. The IPCC concluded in 2013 that there is high confidence that downscaling adds value both in regions with highly variable topography and for various small-scale phenomena [13] such as islands and keys.

In addition, the U.S. Army Corps of Engineers (USACE) developed a sea-level calculator [14] that provides a way to visualize SLR scenarios for any tide gauge that is part of NOAA’s National Water Level Observation Network. Using the USACE sea-level change (SLC) curve calculator with the addition of the NOAA [11] (2017) curves, SLR scenarios were computed for Key West, Florida, the closest tidal measuring gauge to Big Pine Key. The revised regional SLR projections were subsequently used to examine changes in topography and the freshwater lens and resultant habitat changes through analysis of Sea Level Affecting Marsh Model (SLAMM v. 6.4) results [15].

The SLAMM simulates the dominant processes involved in wetland conversions and shoreline modifications during long-term sea-level rise [15]. The model runs using very detailed topographic data. The SLAMM tool addresses various wetland scenarios, including inundation, erosion, over wash, saturation, and salinity. It incorporates sedimentation and accretion rates, standard coastal wetland classes, and provides options for computing erosion. The SLAMM tool then provides acreage of various vegetation and marine communities expected to be available at various levels of SLR on the islands. The communities are then categorized as developed dry land, undeveloped dry land, mangrove and estuaries. This model has been funded, vetted, approved, and applied to more than 100 USFWS National Wildlife Refuges in Regions 4, 5, and 8. The latest SLAMM 6.4 is USFWS sponsored and includes roads and infrastructure. Other users of this model include the U.S. Environmental Protection Agency (EPA), USGS, The Nature Conservancy (TNC), and the National Wildlife Federation (NWF).

### 2.3. Additional Stressors

In addition to information on sea-level rise and habitat change, we examined secondary information about additional stressors, including projected temperature changes, changes in precipitation patterns, changes in tropical system intensity trends, and future urban water supply demands. Using the Coupled Model Intercomparison Project Phase 5 (CMIP5) model, NOAA 2017 [11] revised models to include thermal expansion, ocean dynamics, and glacier mass changes, which increased projected SLR levels. In the meantime, projected temperature changes, changes in precipitation patterns, and changes in tropical system intensity trends were examined through the National Climate Assessment [16]. Finally, future urban water supply demands were characterized through the Water 2070 Technical Report’s methodology to estimate water demand scenarios [17]. This approach was grounded in work previously completed by the University of Florida.

### 2.4. Stakeholder Engagement Workshops

We also examined the utility of considering additional secondary information as part of an integrated effort to connect current knowledge about fish, wildlife, plants, and the habitats of all three to regional climate change projections. A pair of stakeholder-driven workshops (described in the Study Site section) held in 2017 focused on identifying impacts to species under different SLR scenarios to inform adaptation planning, including prioritization of actions, examining risks, and examining adaptive capacity and governance issues [18]. We examined three types of findings from these workshops in the context of developing a more holistic analysis, including overarching actions considered for multiple species and species- specific actions that will benefit the Florida Key deer.

## 3. Results

### 3.1. Inventory of Existing Conditions

#### 3.1.1. Topography and Freshwater Lens

**Figure 1.**
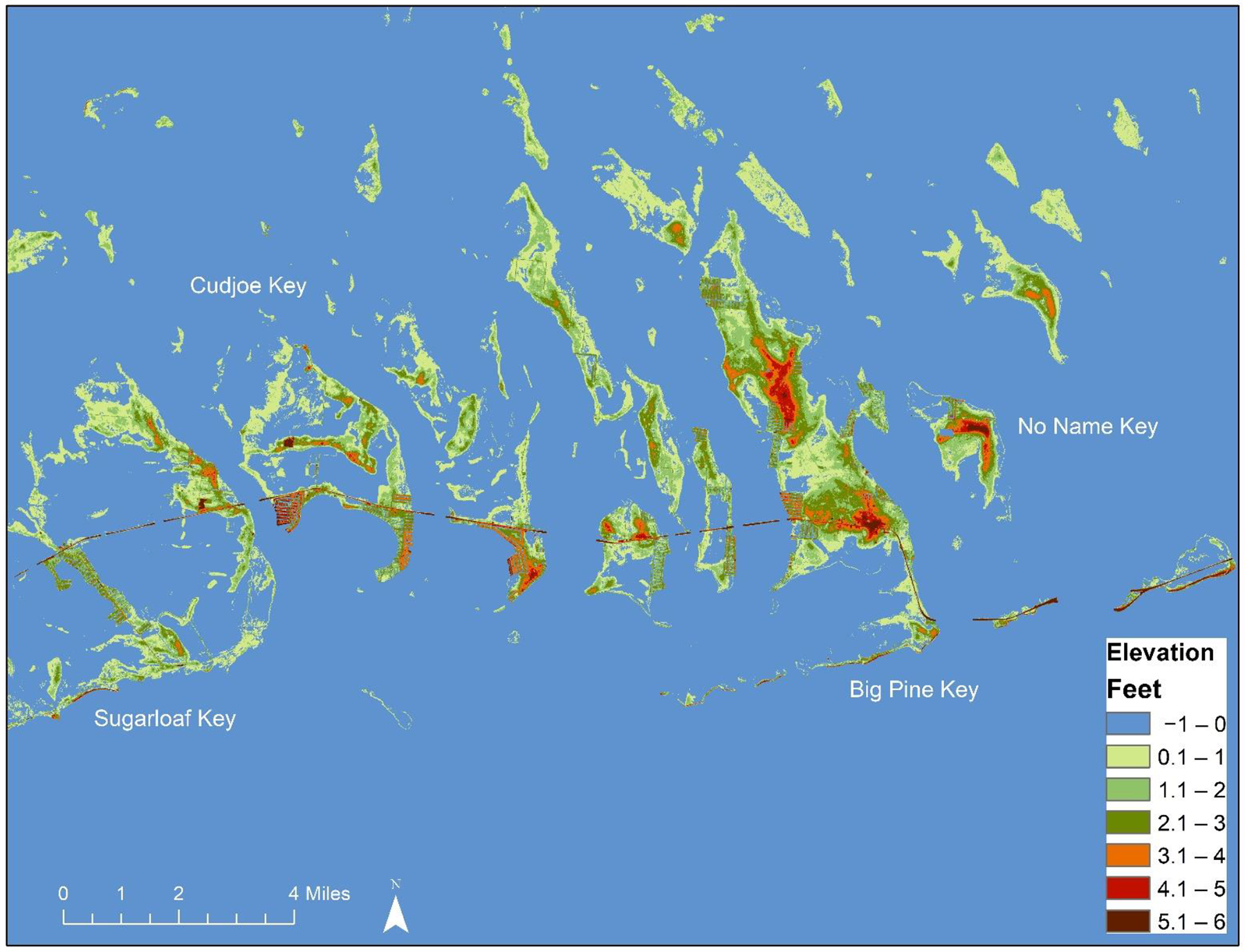
Current elevations of the middle and lower Keys (from [15]).

The majority of Big Pine Key, along with the other middle and lower Keys, has an elevation less than 1.2 m with only 10–20% of the islands land mass over 1.2 m elevation (Figure 1).

A unique characteristic of the Florida Keys is the existence of a freshwater lens below the islands that is critical for humans, wildlife, and habitats. Below the shallow soil surface, which is less than 20 cm deep, is limestone rock [19]. The principal freshwater-bearing unit underlying Big Pine Key is a layer of oolitic limestone averaging 5.8 m in thickness. Below that is a shallow layer (or lens) of fresh water. The lenses are not a large volume of freshwater, so only a small amount of freshwater in the lenses can be removed before it is replaced by saltwater intrusion [20]. The fresh groundwater is less dense than the underlying saltwater, causing it to float on top of the saltwater and reach up into the limestone rock formations and solution holes of the island providing fresh water to root zones and terrestrial species. The areal extent and depth configuration of the freshwater lenses are affected by tidal fluctuations and fluctuate in response to rainfall, evapotranspiration, lateral and vertical losses, and pumpage from local wells. The freshwater exists in two separate lenses on Big Pine Key, one in the northern half of the island and one in the southern half. The slightly larger north lens is separated from the south lens by a low-lying land area 0.3–0.9 m above mean sea level.

#### 3.1.2. Ground Surface Inundation versus Root Zone Salinization

**Figure 2.**
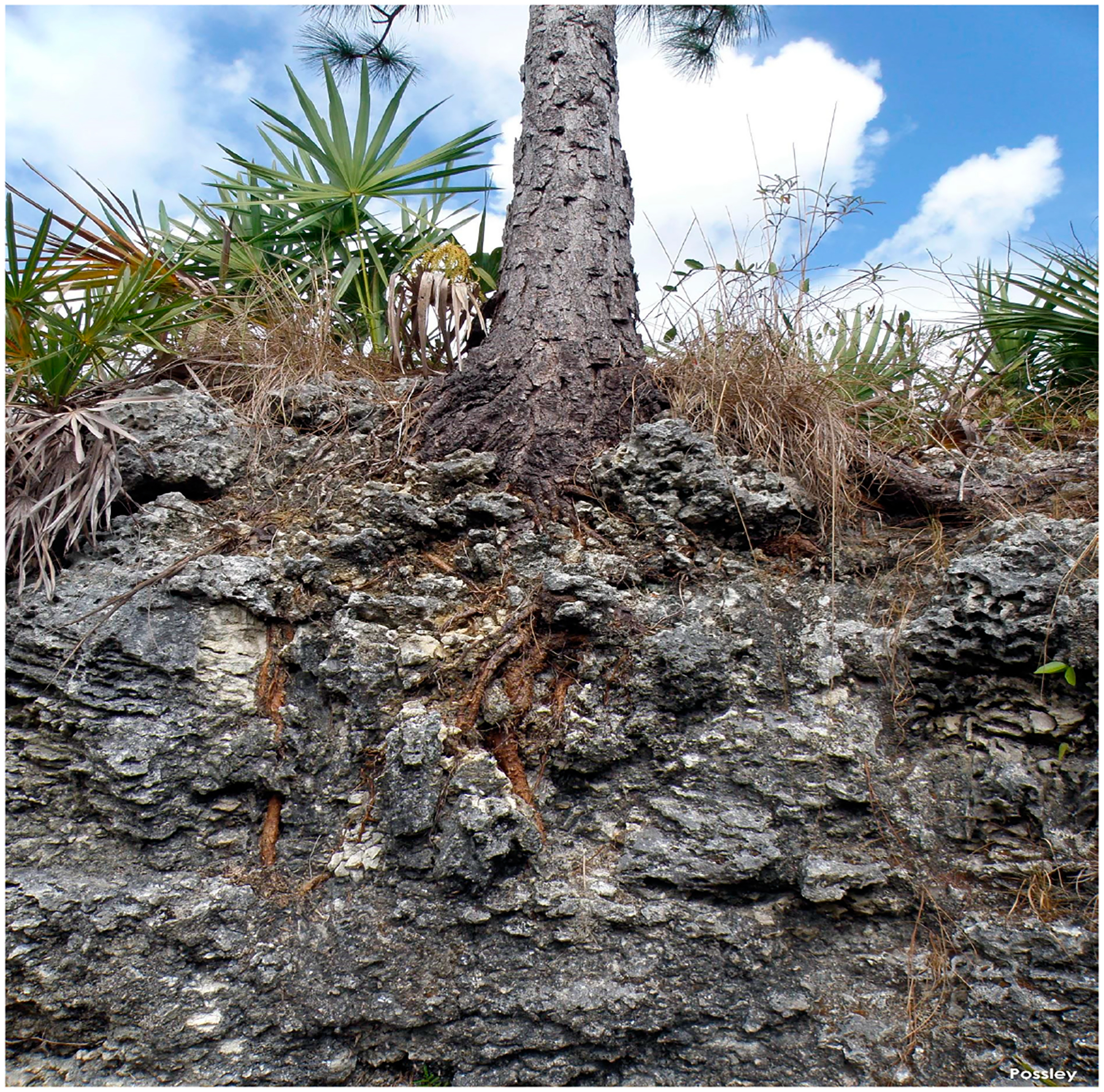
Slash pine (*Pinus elliotti*) roots indicating the root zone in Florida Keys limestone of 0.5–0.9 m depth. (Photo credit to Jennifer Possley).

Saha et al. [21] recognized that South Florida’s low-lying coasts have plant communities organized along a mild gradient in elevation, from mangroves at sea level up to salinity-intolerant coastal hardwood hammocks on localized elevations generally less than 1.8 m above sea level [22]. The most productive island forests (pine rocklands) are situated at slightly higher elevations in the interior of Big Pine Key and No Name Key with the majority of land elevation below 0.9–1.2 m, where they are currently least likely to be regularly inundated by spring tides or storm surges. Past Florida Keys studies have focused primarily on ground surface inundation of ocean water to determine effects to vegetation. Hardwood species of inland hammocks are deep rooted (Figure 2) and use fresh groundwater from the freshwater lens for hydration [21,23].

**Figure 3.**
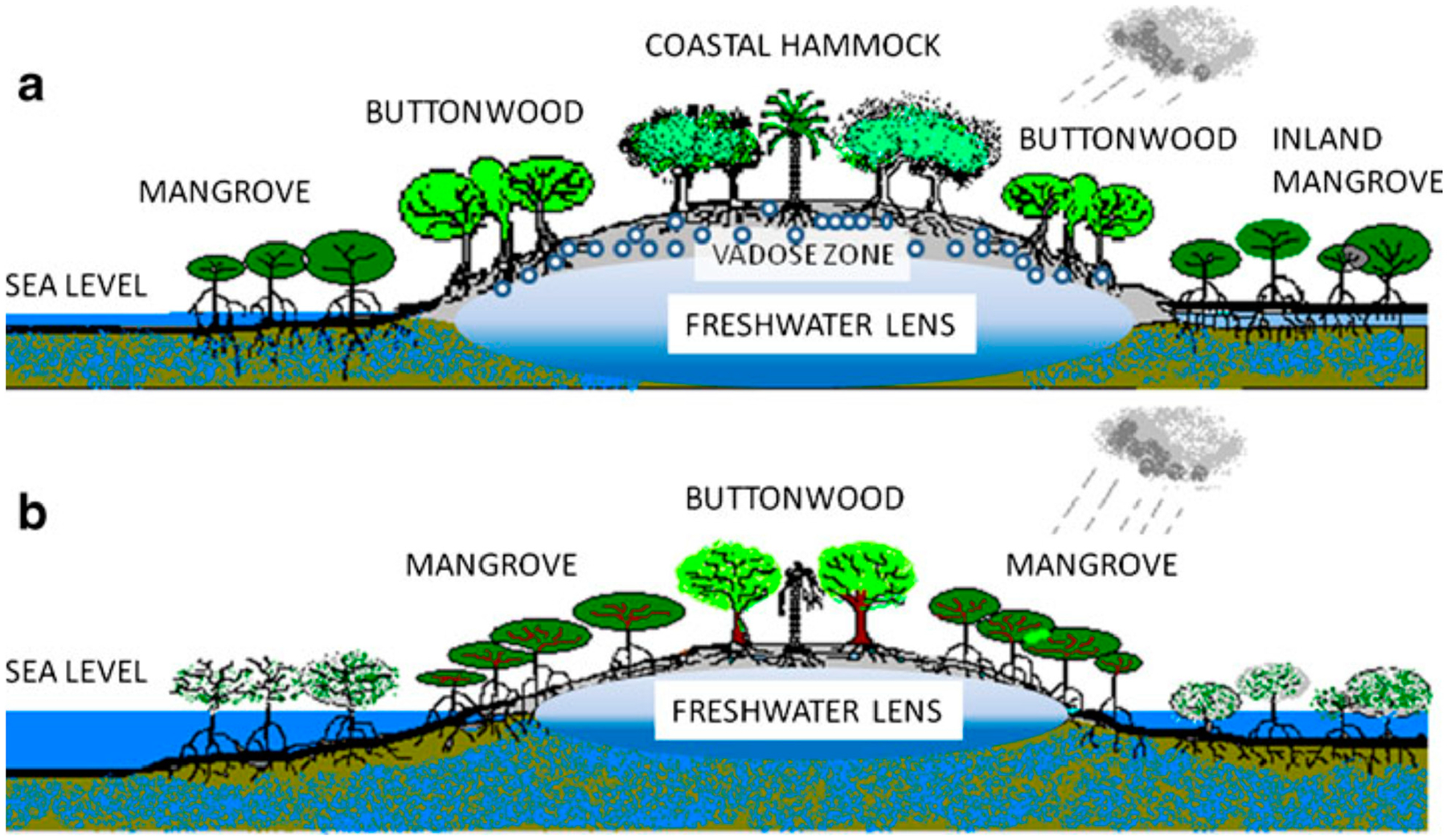
A sketch of a coastal hammock (**a**) on an elevated rise flanked by buttonwood forests at intermediate elevations and mangrove forests at sea level. Also shown is the vadose zone (zone from the ground to the water table), freshwater lens (where shading indicates increasing brackishness towards the bottom of lens), and seawater. Bottom sketch (**b**) shows a rise in sea level that decreases the volume available to hold freshwater in the freshwater lens, with consequent mortality of coastal hammocks and the upward migration of buttonwoods and mangroves. Elevation exaggerated in illustration to indicate water pools (reproduced from [21] with permission).

This water zone used by roots is called the vadose zone. Sternberg and Swart [24] showed that the hardwood hammock species in the Florida Keys growing next to coastal saline waters had isotopic signatures similar to freshwater, indicating that roots of hardwood species extracted freshwater from the soil and vadose zone at the top of the freshwater lens (Figure 3).

Ross et al. [19] suggest that shrinking of the freshwater lens in the coastal uplands of the Everglades is a likely cause of initial vegetation change because of SLR. These long-term consequences of SLR will prove detrimental to buttonwood and hardwood hammock habitats, important to many endemic species. Decline in capacity of freshwater storage and resources will be made worse with the additive effects of storm surges and unusually high tides. NOAA [12] recommends adding 0.3 m to SLR scenarios to obtain a better understanding of how high tides exacerbate the rising sea levels.

#### 3.1.3. Sea-Level Rise to Date

**Figure 4.**
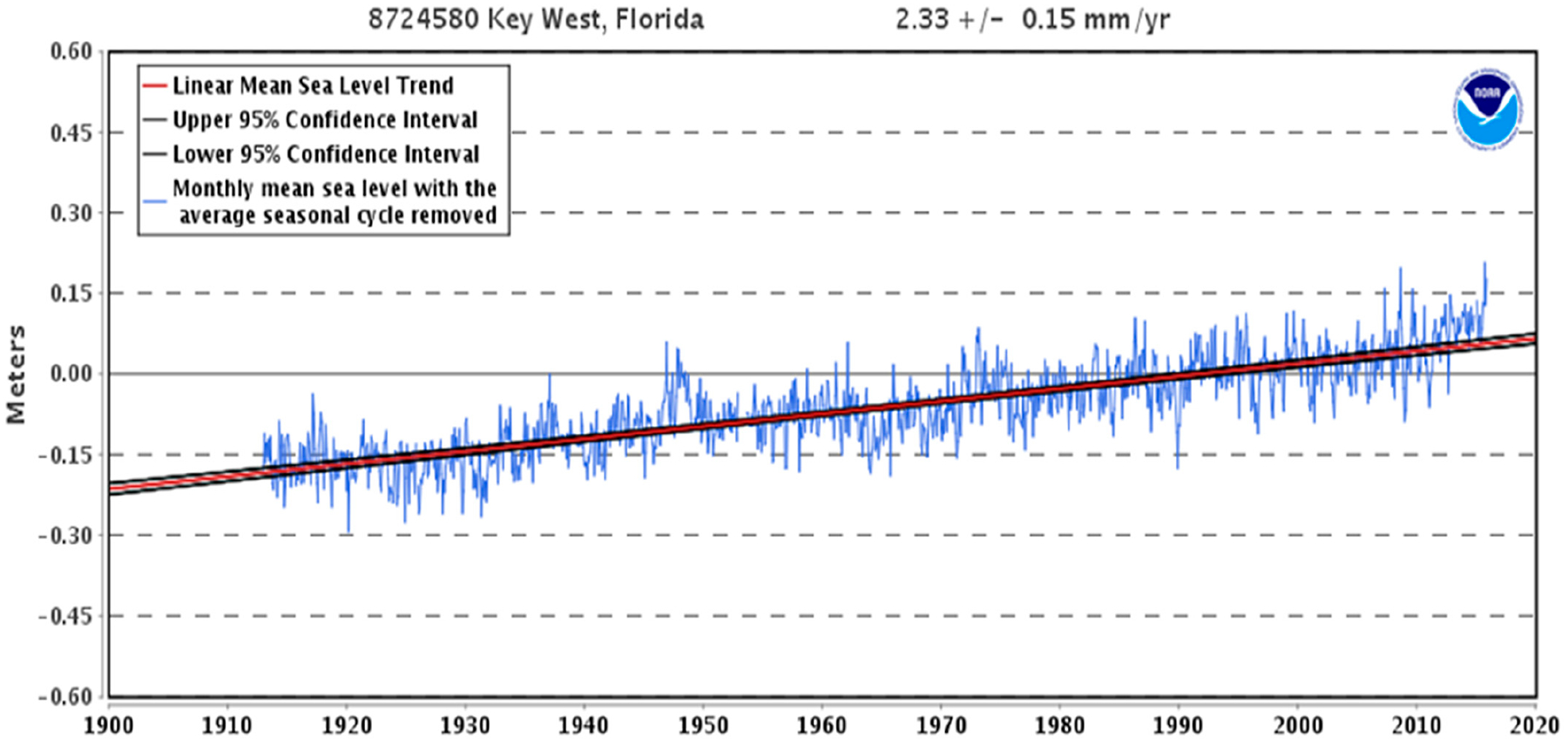
Historical sea levels in Key West show a rather linear trend of average sea levels from the early 1900s. SLR rates began to accelerate in the early 2000s as shown on the right side (from [25]).

According to the NOAA Station KYWF1–8724580 (Key West ocean data buoy), the rate of SLR in the Florida Keys had been an average rate of 0.23 cm/yr (Figure 4) prior to the last decade [25]. The NOAA plot shows monthly mean sea level without regular seasonal fluctuations due to coastal ocean temperatures, salinities, winds, atmospheric pressures, and ocean currents. The long-term linear trend is also shown, including its 95% confidence interval. The plotted values are relative to the most recent Mean Sea Level datum. It is noticeable in Figure 4 that the sea level rose around 1945 (3-year duration) and again in the early 1970s (5-year duration). The difference in those trends, and the current acceleration trend, is that greenhouse gases (GHGs) are presently rising at a faster rate by 2 ppm yr^−1^ than the1940s and 1970s.

**Figure 5.**
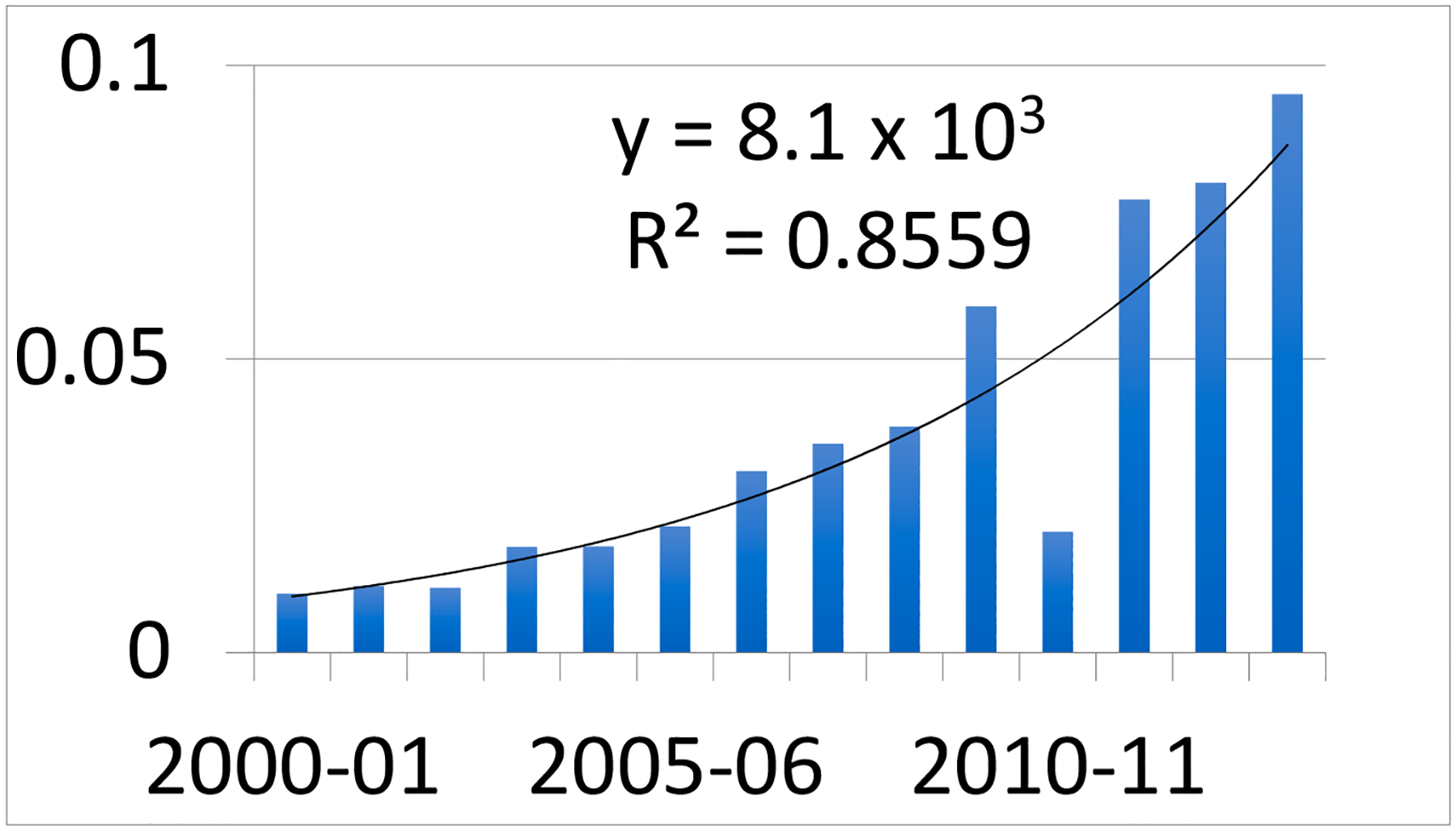
The rate of SLR began its exponential (accelerated) rise in the early 2000s (modified from [18]).

In the early 2000s, SLR began to accelerate exponentially (Figure 5) and was estimated at 0.76 cm yr^−1^ in 2016 [18], an increase of 0.53 cm yr^−1^. The rate of SLR began to increase at a faster rate in the early 2000s, about 75–100 years after GHG emissions began to rapidly increase due to a “lag effect.”

#### 3.1.4. Historical Impacts to Island Hammock and Pine Forest with Sea-Level Rise

As a string of small islands, SLR continues to change the shape and size of the Florida Keys along with changes in vegetation types and coverage. For example, SLR has led to the decline in pine trees and pine ecosystems in the Florida Keys coastal forest habitat [26].

**Figure 6.**
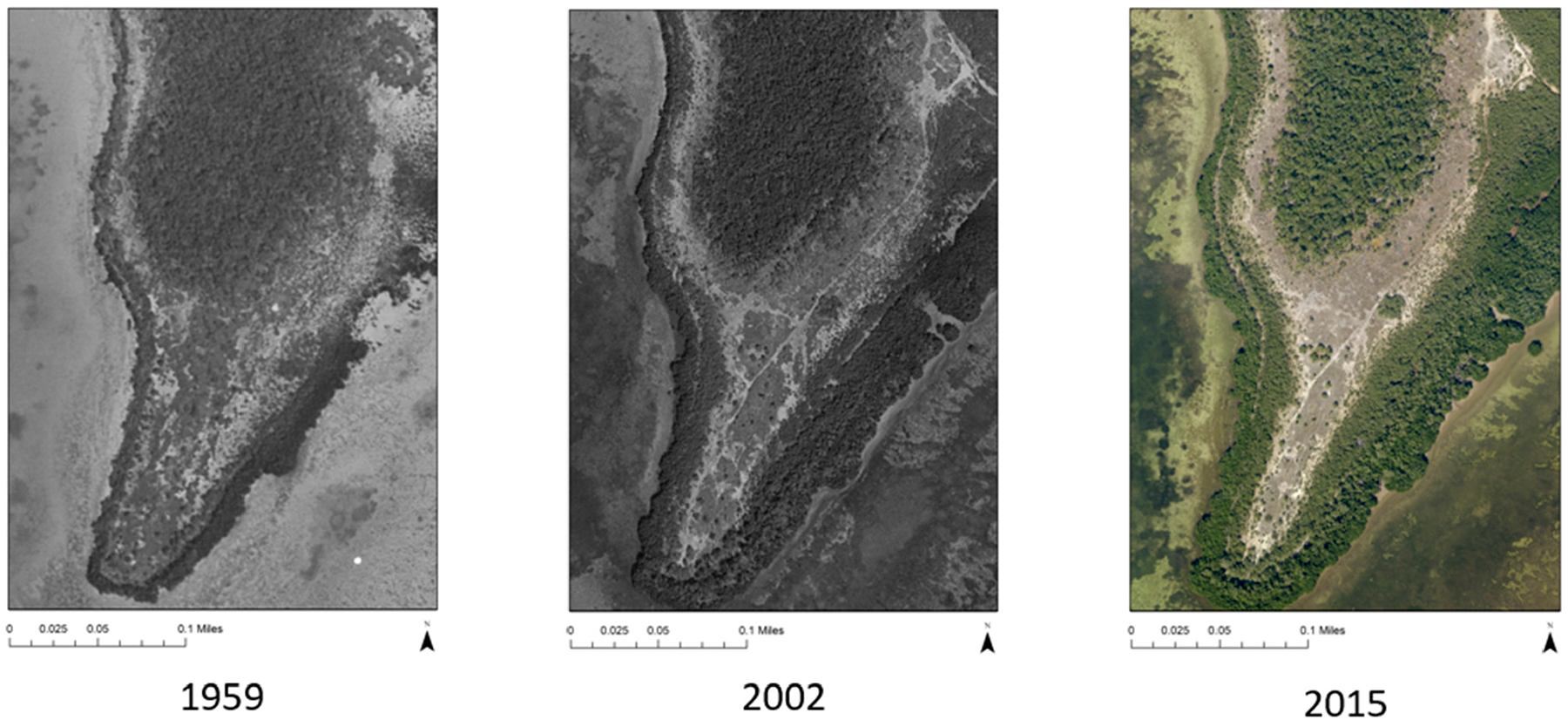
Historical image of SLR in southern No Name Key-Historical aerial photographs for southwestern No Name Key. In the photographs, the dark outline areas of the peninsula are mangroves, and the internal dark area is pine rockland and tropical hardwood hammock. The 1959–2015 photographs depict an encroaching mangrove zone from 15.2–17.8 cm of SLR (from [28]).

The decline of pine forests on the Florida Keys were documented by Alexander and Dickson III [27]. Following up on local homesteaders’ accounts of live slash pine trees bordering a remote mangrove swamp on the north end of Key Largo, Alexander and his students found only remains of dead trees. Alexander later attributed the forest’s demise to salinization of the groundwater in response to SLR. More recently, Ross et al. [19] stated that the area of pine forest on Sugarloaf Key declined from an initial 217 acres (88 ha; furthest extent of pine remains) before 1935 to 30 ha by 1991. Ross et al. [19] suggest that shrinking of the freshwater lens in the coastal uplands of the Everglades is a likely cause of initial vegetation change because of SLR. Figure 6 shows an example of an area on No Name Key (the sparsely inhabited island next to Big Pine Key) with primary upland habitat. Vegetation changes are studied on this island through the use of aerial photographs from 1959, 2002, and 2015. In these images, the vegetation changes to increased mangrove and estuary around the perimeter with shrinking hardwood hammock and pine trees as illustrated in the center of the photographs.

#### 3.1.5. Threatened and Endangered Species

The Benedict et al. [18] workshop summary provides a recent introduction to the 21 threatened or endangered species (and habitats) in the Florida Keys, including Big Pine Key. Benedict et al. [18] used a stakeholder-based series of focused workshops to systematically examine 21 species (and habitats) in the Florida Keys in the context of climate change and other threats to provide guidance for developing adaptation strategies and prioritizing management actions. For three species, the Lower Keys Marsh rabbit (*Sylvilagus palustris hefneri*), the Key tree-cactus (*Pilosocereus robinii*), and the Miami blue butterfly (*Cyclargus thomasi bethunebakeri*), they also examined barriers to implementing climate adaptation actions and how to “manage for change” (i.e., the ongoing natural resource management efforts with climate-driven changes in mind; [29]) for those species.

The Florida Key deer, a subspecies of the white-tailed deer, was also a discussion focus of the 2017 workshop. The Key deer population range has been reduced to the middle Florida Keys. Florida Key deer have genetic, physical, and behavioral differences from mainland deer [30–33]. Geographically constrained to approximately 20 islands, anthropogenic and natural stressors have caused declining populations over the years. Historically (1950s), the subspecies was hunted to near extinction in the keys. As a result, the Florida Key deer was listed as federally endangered in 1967 [34] and remains on the endangered list to date. The main anthropogenic source of deer mortality today is by car strikes.

Although generalists in mainly upland habitat utilization, this habitat is declining due to human development, saltwater intrusion in upland root zones, and the encroachment of salt tolerant mangroves. The availability of freshwater resources is becoming an even more important environmental stressor, including risks associated with major salinization events, such as tropical cyclone storm surge and rising high tide events due to SLR. For example, Benedict et al. [18] estimated that a 0.3 m SLR resulted in a 37% loss in freshwater resources, and complete loss of freshwater with a 0.6 m rise in the middle Keys.

### 3.2. Sea-Level Scenarios and Resultant Habitat Impacts

#### 3.2.1. SLR Scenarios for Big Pine Key, Florida

The NOAA [11] report provided a scientific and modeling review of the IPCC (2013) and the National Climate Assessment-3 [16]. Naming conventions were changed from previous modeling endeavors (see Appendix A on summary of 2017 climate scenario development in the back of this report). The global mean sea-level rise (GMSL) was adjusted to account for key factors important at regional scales including: (1) shifts in oceanographic factors such as circulation patterns; (2) changes in Earth’s gravitational field and rotation, and the flexure of the crust and upper mantle due to melting of land-based ice; (3) vertical land movement (subsidence or uplift) due to glacial isostatic adjustment (which also changes Earth’s gravitational field and rotation, as well as the overall shape of the ocean basin); (4) sediment compaction; (5) groundwater and fossil fuel withdrawals; and (6) other non-climatic factors.

**Figure 7.**
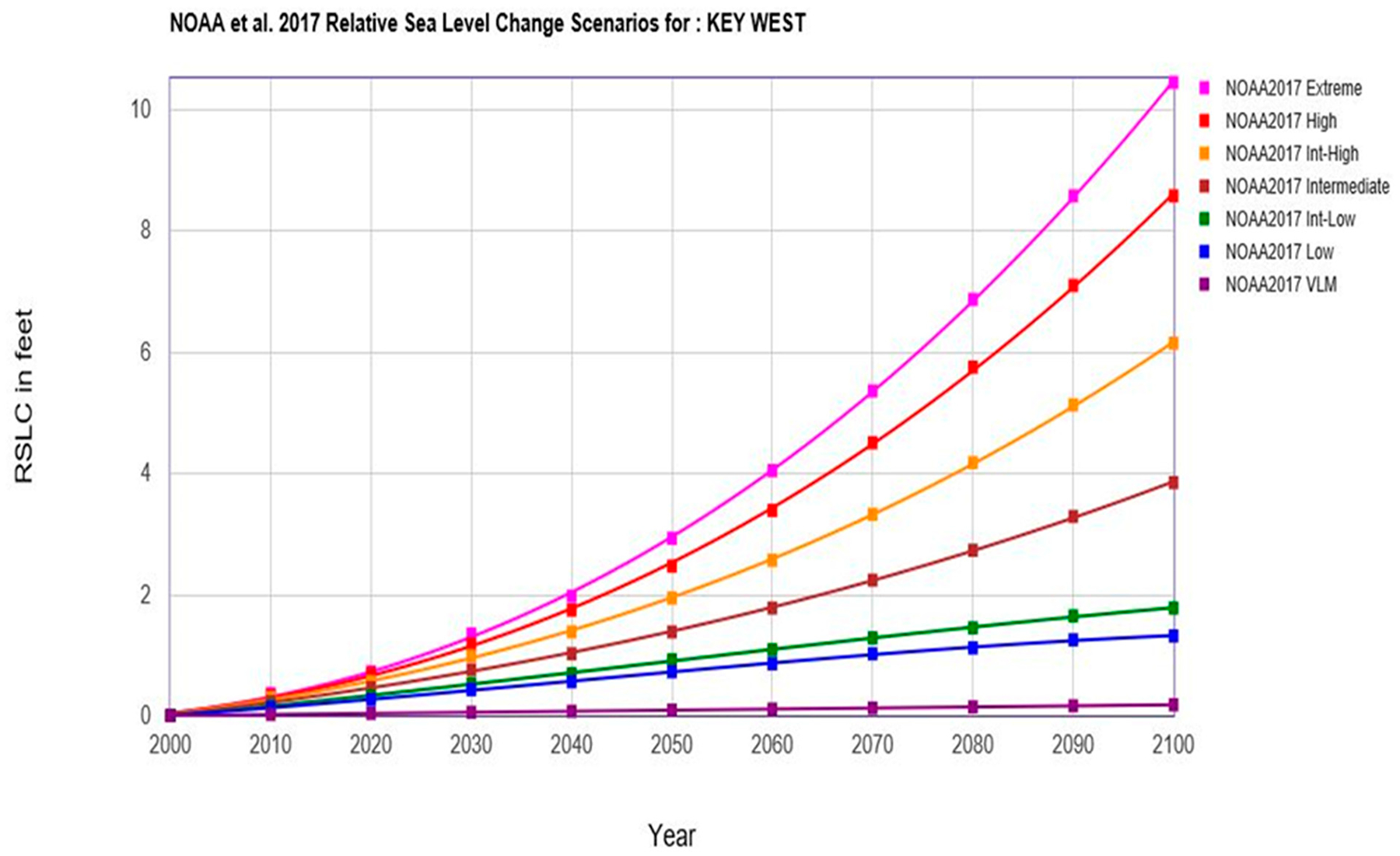
The NOAA VLM (2000 extrapolation), low, intermediate low, intermediate (GHG decreases in 2060), intermediate-high (GHG decreases in 2080), high (“business as usual” with no decreases in GHG), and extreme (rapid sheet ice melt) scenarios for sea-level rise (from [11]).

The top line in Figure 7 is the 2017 NOAA Extreme rate that represents accelerated ice sheet melt that has been recently documented [35]. The next red line is the 2017 NOAA High rate that represents “business as usual,” or GHG emissions of 2 ppm yr^−1^ or greater. This scenario describes GHG emissions that do not decrease over time. This line incorporates some melting of the Antarctic ice shelf (95% confidence interval); the polar ice sheet melt was unknown during the IPCC 2013 [13] scenario model runs and thus this represents new science captured within the NOAA 2017 [11] report. The orange line is the 2018 USACE High scenario and is also “business-as-usual” where GHG emissions continue to rise until 2080 (95% confidence interval) but without the input of any polar ice sheet melt. The crimson line is the 2017 NOAA Intermediate High rate that shows GHGs starting to decrease in 2060. The next two lines, 2017 NOAA Intermediate Low and 2017 NOAA Low, have current GHG emissions decreasing by 2040 and 2020, respectively. The last line indicates a 2017 NOAA mathematical extrapolation from GHG emissions in 2000 with no other variables.

**Figure 8.**
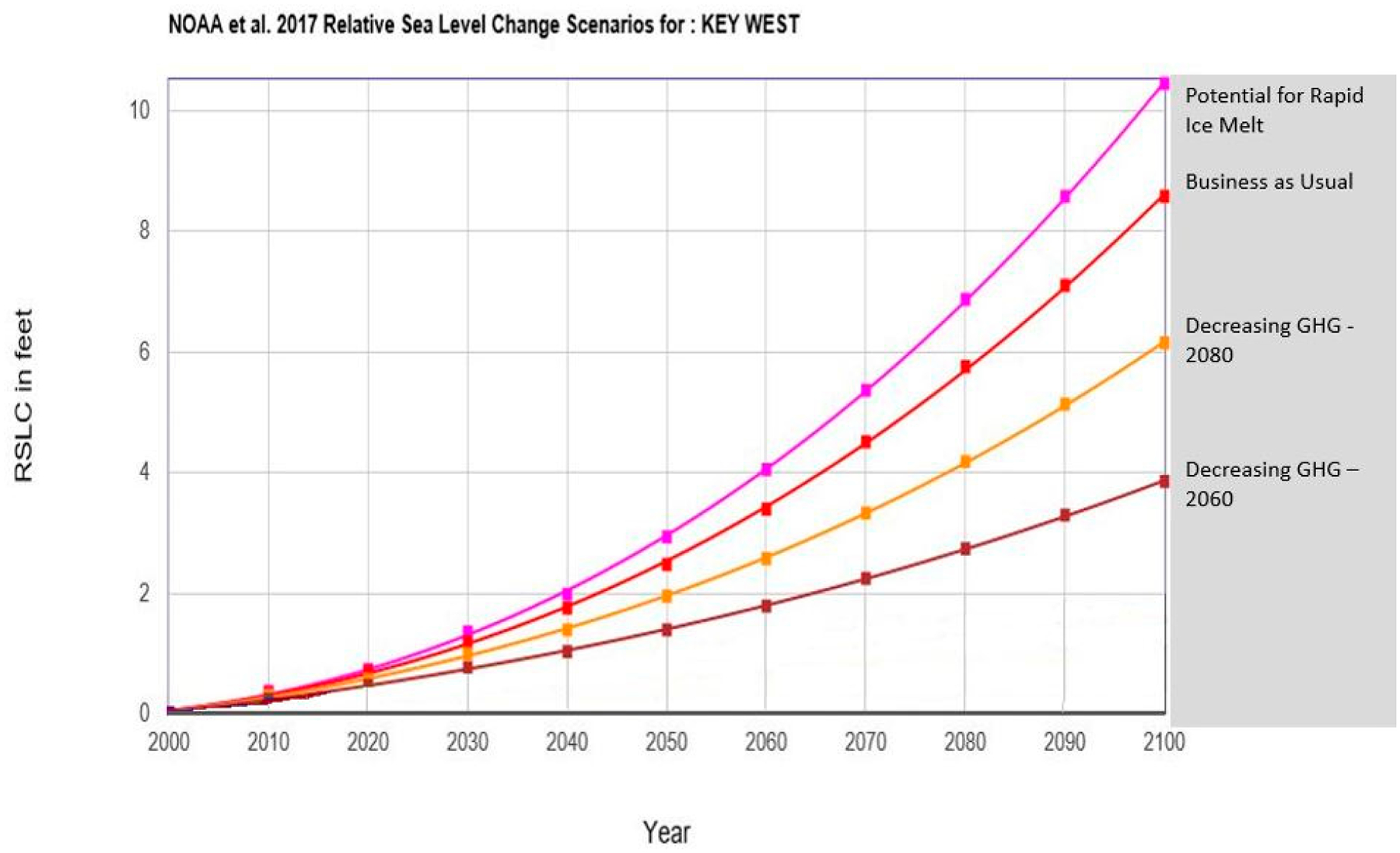
USACE relative sea-level change (RSLC) curve calculator incorporating the NOAA 2017 [11] update of the IPCC [13] (2013) sea-level change curves at Key West, Florida [14,36]. This figure removes the lowest three IPCC scenarios due to NOAA 2017 [11] analysis that these either have been, or will be, exceeded with 96–100% probability (modified from [28]).

A common misconception is that this set of scenarios is based on a single data set capturing varying degrees of uncertainty. In reality, each line in the scenario graph in Figure 7 was developed with a different data set, each primarily dependent on the amount of GHG emissions that are currently being released coupled with a projection of continued, decreased, or increased releases over time. Thus, the set of scenarios, that include several scenario lines, are not intended to provide a probabilistic prediction of future changes. Rather, scenarios are intended to describe plausible conditions that support decision-making under uncertainty, given specific assumptions about which SLR science to include in a given risk assessment. It is critical that decision makers are made aware of this misconception. To assist in this understanding, the scientific nomenclature of the 2017 NOAA scenario lines were re-labeled for decision makers. This increases the understanding on what is driving that particular scenario (Figure 8). Furthermore, as the probability of exceeding the lowest two NOAA 2017 [11] scenarios (2017 NOAA Intermediate Low and 2017 NOAA Low) was 96%, these two scenarios, along with the 2017 NOAA 2000 extrapolation scenario, are eliminated from the scenarios presented here (Figure 8).

#### 3.2.2. Sea-Level Rise Impacts

The original 2013 sea-level calculator for the Florida Keys was updated with NOAA and USGS 2017 [11] revisions to the 2013 Intergovernmental Panel on Climate Change (IPCC) [13] climate projections building upon newer information.

**Figure 9.**
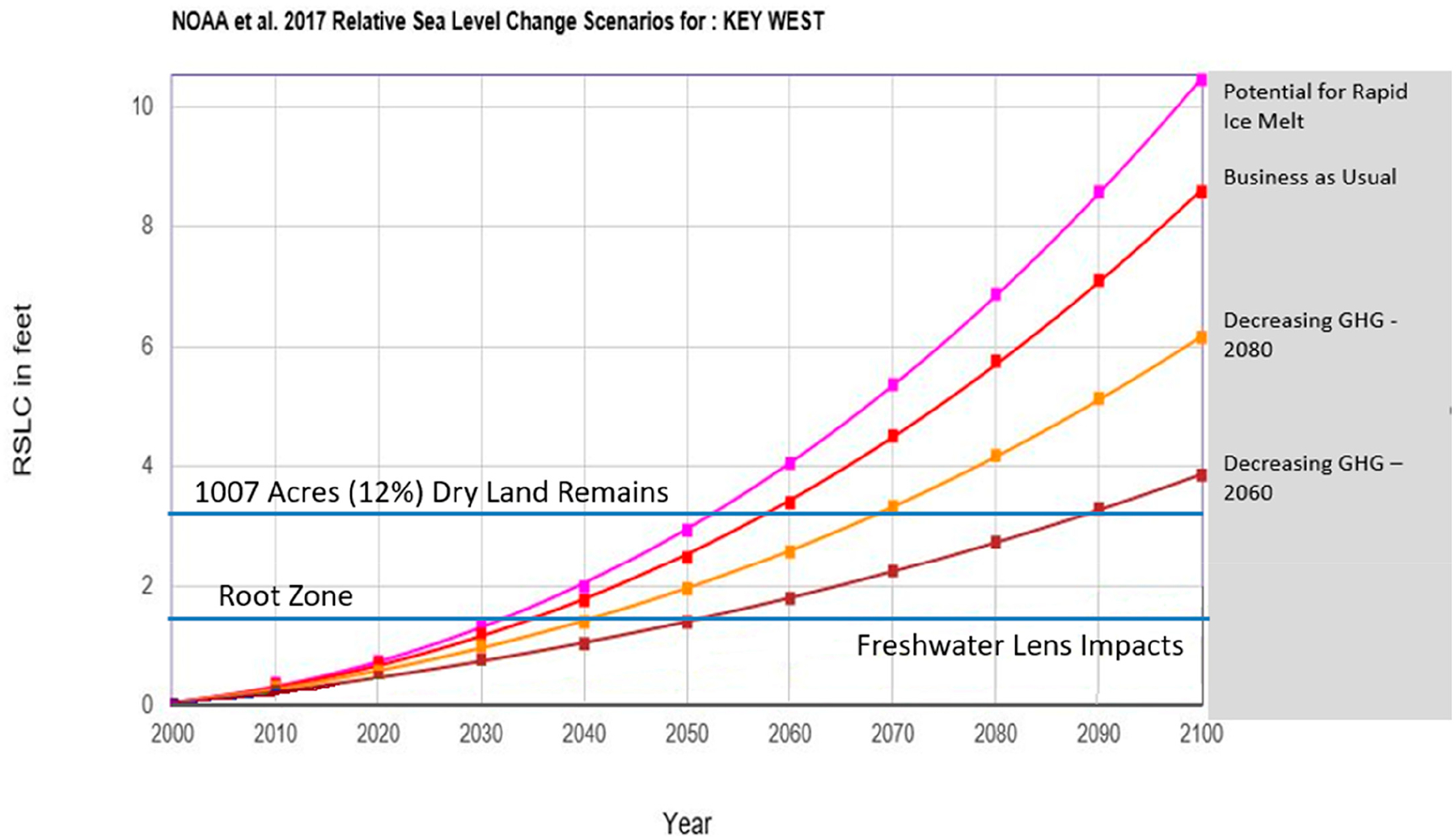
Relative sea-level change (RSLC) curve calculator [14] illustrating expected impacts to Florida Keys upland habitat and the freshwater lens. The dark green (top) horizontal line marks the 0.9 m elevation where only 408 ha, or 12% of dry land (developed and undeveloped) remains, on Big Pine Key. The light green (bottom) horizontal line indicates the root zone depth within the highest elevations where plants will be impacted by SLR [28]. Root zone and freshwater lens impacts will occur prior to these horizontal lines for all vegetation below 0.9m elevation. This line is only indicating the SLR at which the last remaining uplands (>0.9 m elevation) will begin to be impacted (modified from [28]).

As decision makers grapple with understanding scenario graphs, it is apparent that highlighting important environmental conditions, or tipping points, is necessary. Thus, green horizontal lines are added to the scenarios in Figure 9 that include the point at which the root zone, freshwater lens, and diminished available dry habitat become impacted with saltwater in the future.

The effects of SLR on the Florida Keys are exacerbated due to a combination of topography and reliance of plants and animals on the freshwater lens (Figure 3). Because there is limited habitat or dry undeveloped land above 0.9 m elevation on Big Pine Key (243 ha) or 7% of dry undeveloped land remaining), there is little benefit to analyzing SLR scenario curves above that elevation (Figure 9; top horizontal line). Once sea levels rise above the island, the rate of SLR is inconsequential because the island will be predominately mangrove, estuary, and open water. In contrast, the root zone (0.46 m below the surface) for the slash pine community and other upland vegetation [19] will start to be severely impacted well before surface inundation by SLR (Figure 9; bottom horizontal line). This will reduce habitat for the Florida Key deer along with necessary fresh water drinking sources, thus leading to further declines in the deer population. While saltwater is already intruding along the island coasts and the freshwater lens, saltwater will begin to negatively affect the root zone of the island’s last upland vegetation between the years of 2040 and 2060 under all four scenarios. The island will be mostly underwater between the years of 2060 to 2080 in all recommended SLR scenarios.

During the period from now out to 2040–2050, SLR will likely move between various scenario lines. There is higher uncertainty from 2020–2050 due to global model uncertainty of SLR acceleration rates [11] based on predicted human behavior. Beyond 2050, it is likely that the SLR rate of increase will become more apparent. There is less uncertainty in SLR projections from 2050–2100 after the initial acceleration (now through 2040–2050) has been completely defined. Therefore, the resulting SLR “spread” at 2021 is not an indication of high uncertainty, but rather represents a forecast range of SLR due to varying GHG emission rates.

The most noticeable impact from SLR is the increasing frequency of high tide flooding (HTF), thus examining future projections (i.e., 2030 and 2050) provides vital information for addressing coastal flooding impacts [12]. The NOAA tidal gauge data used to measure rapid increases in coastal flood risk along U.S. coastlines due to SLR rise can provide insights into changes in frequency of high tide flooding (an additional 0.30 m). The 2020 NOAA report [12] used the 2017 SLR scenarios and indicated that today’s national HTF frequency at five days per year (national median) will increase to 7–15 days per year by 2030 and 25–75 days per year by 2050, with much higher rates in many coastal locations. Combining a tide and/or tidal flooding elevation with SLR projections causes impacts to root zones, freshwater lens, and upland vegetation communities much earlier in time than just SLR projection data alone. Table 1 indicates that inundation and pre-inundation impacts to the Florida Keys happens 10–20 years sooner depending on the scenario.

#### 3.2.3. Habitat Change Impacts

**Figure 10.**
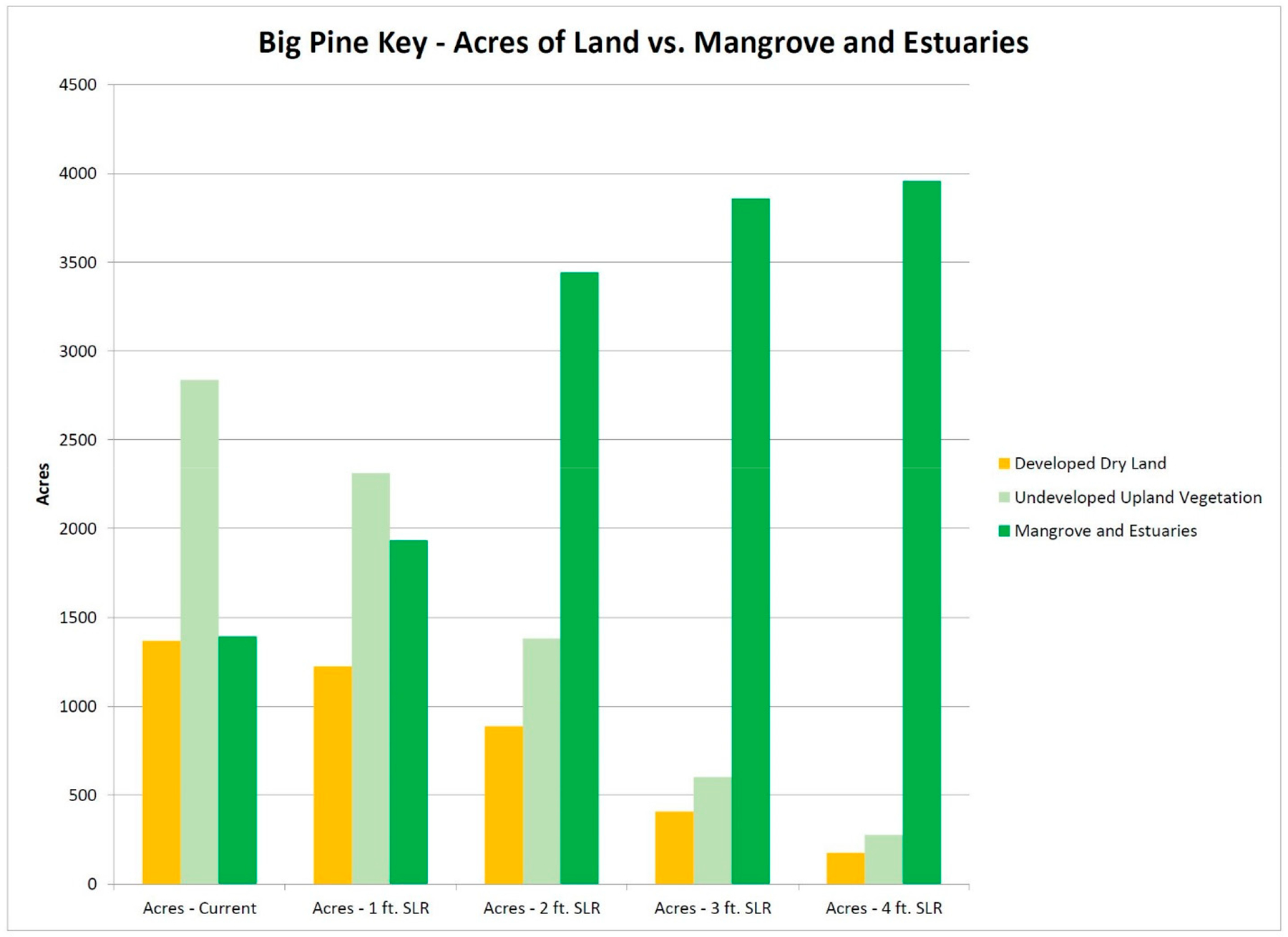
Developed dry land (orange) and undeveloped upland vegetation (light green) on Big Pine Key will decrease in availability due to SLR with as little as 0.3 m of SLR while coverage of mangroves and estuarine waters (dark green) becomes the majority of habitat with SLR of 0.6 m or more (from [28]).

Projected changes in land cover conditions with 0.3 m, 0.6 m, 0.9 m, and 1.2 m of SLR are shown in Figure 10. The main changes are conversion of upland vegetation to mangroves, estuarine, and open ocean waters from undeveloped hardwood hammocks and upland pine communities. The SLAMM v. 6.4 modeling shows that by 0.9 m of SLR most, if not all, of the upland slash pine community will be dead (but trunks usually remain) and mangroves will have encroached almost completely into those communities that are so necessary for species such as the Florida Key deer. Thus, the deer population is expected to decrease and become displaced due to lack of any habitat. Urbanization also decreases as SLR increases because of surface inundation of seawater and the lack of available dry land. Upland habitat and available dry land decreases while mangroves, if able to keep up with the rate of SLR, will become dominant vegetation.

Table 2 presents the remaining developed dry land and undeveloped dry land under different SLR conditions. Dry land will decrease in availability with as little as 0.3 m of SLR while mangroves become the predominant vegetation community with SLR of 0.6 m. By 0.9 m of SLR, mangroves will have encroached almost completely into upland habitat on Big Pine and No Name Keys. Only 7% of undeveloped dry land (potential terrestrial habitat) and 5% of urban areas remains with 0.9 m of SLR.

### 3.3. Additional Stressors

#### 3.3.1. Projected Temperature Changes

Average temperatures in the Florida Keys have risen 1.0–1.6 °C since the 1900–1960 period [37]. Projected increases in South Florida’s average annual temperatures are an additional +0.5 to +1.6 °C during the 2035–2065 period and up to +1.6 to +2.7 °C during the 2070–2100 period depending on the current GHG emissions rate and the region within Florida [37]. State-wide temperature increases will change levels of humidity and rates of evapotranspiration leading to changes in vegetation growth, seasons, and location. As an example, increasing temperatures can affect the sex ratio of some species, especially reptiles such as sea turtles, leading to a decline in species population if the sex ratio between the number of males versus the number of females becomes large. If sea turtle eggs incubate below 28 °C, turtle hatchlings will be male; if eggs incubate above 31 °C, the hatchlings will be female [38].

#### 3.3.2. Changes in Precipitation Patterns

Annual precipitation has increased by 5% since 1900 in the Florida Keys [37]. Since the 1970s, heavy downpours have increased in frequency and intensity by 27% and are increasing island flooding along the coastal beaches and in low-lying areas. Model simulations using the more recent CMIP5 predicts changes in seasonal precipitation for the Florida Keys with increases in dry season rainfall up to 20% and decreases in wet season rainfall up to 30% [11]. A decrease in wet season rainfall will lead to lower water levels and increased droughts during a time that plants are water-dependent for growing and flowering and wetland bird species are foraging. The change in timing of rainfall will stress ecosystems and cause changes in vegetation types. An increase in dry season rainfall will increase water levels and hydroperiods during the important time of year when many birds are preparing to breed and nest, migratory birds are stopping over to forage, alligators are preparing nesting holes, and plants are becoming more dormant.

#### 3.3.3. Tropical System Intensity Trends

Current models are in agreement regarding changes in tropical storm and hurricane wind and rainfall events with 20% increases in both rainfall rates and wind speeds expected near the center of storms [11]. These increases are linked, in part, to higher sea-surface temperatures in the region where Atlantic hurricanes form and move through. It is important to note that other factors influence the frequency and intensity of hurricanes. Natural variability of the Atlantic Multi-decadal Oscillation (AMO), El Niño Southern Oscillation (ENSO), human-induced emissions, and particulate pollution all influence warming or cooling of sea-surface temperatures which drives the annual activity of tropical storms and hurricanes [28]. Increases in storm rainfall rates and wind speeds can exacerbate the effects of negative impacts to vegetation communities, effects of storm surge, and physical danger to terrestrial species.

#### 3.3.4. Future Urban Water Supply Demands

The Water 2070 Technical Report [17] developed land-use patterns most likely to occur if 2070 population projections of roughly 15 million additional residents above 2010 levels are met and counties continue to develop at 2010 development densities. Overall, Monroe County, which includes all of the Florida Keys, is expected to grow at a medium rate of 5.99%, with a concomitant increase in water demand (development plus agriculture) from 81.8 to 87.4 million liters day^−1^, or a 6.9% increase over 2010 [17].

### 3.4. Stakeholder Engagement Workshops

In 2017, a group of stakeholders (Florida Keys Case Study 97) met to discuss incorporating climate change considerations into conservation planning and actions for 98 threatened and endangered species in the Florida Keys (Federal Keys Adaptation project; [18]). Workshop participants worked in groups to brainstorm potential SLR consequences at several intervals and developed a matrix of adaptation strategy options. The group identified a number of coarse filter (overarching actions considered for multiple species), fine filter (species specific in situ actions), and ex situ actions that will benefit the Florida Key deer [18]. Many important decisions and recommendations were vetted for various plant and animal species, including the Florida Key deer, during the workshop, such as:

- There is currently sufficient existing species knowledge to make robust decisions.
- Non-climate stressors (e.g., urban development) can be equally or more important in the short-term when coupled with climate-based stressors, such as storm surge, spring tides, and hurricanes.
- Experienced scientists and managers are needed to interpret models and to use them to help develop adaptation options.
- Priorities should be established based on social and economic values. In some cases, resources can be prioritized for greater species at risk or more socially or economically valuable species.
- Risks have multiple dimensions associated with action and inaction. Each of these should be assessed on a species-by-species basis or based on species with shared vulnerabilities.
- Adaptation is by design an ongoing process. For example, Climate-Smart Conservation [8] emphasizes regularly revisiting goals, objectives, approaches, and strategies.
- If natural resource representatives can combine with representatives from other sectors (e.g., social, economic, and ethics sectors) to develop multi-dimensional adaptation strategies, there will be a greater opportunity to ensure that priorities of natural resource managers are achieved.
- Implementation is difficult, and ways need to be found to move forward with implementing adaptation strategies.

This suite of recommendations represents a holistic characterization of conservation planning and future recommended actions in response to climate change and SLR.

## 4. Discussion

### 4.1. Robustness and Sustainability of Approach

An important finding that is transferable to other locations/species is the value of combining and integrating current state-of-the-science information with rigorously developed local/regional climate projections. Another important finding is the necessity to bring in information on additional (or co-occurring) stressors and social-ecological sustainable information developed through meaningful stakeholder engagement. This approach is also robust for re-applying to the same location/species when new science becomes available. For example, at the time of this study, the NOAA 2017 SLR projections were considered the state-of-the-science. Since this manuscript was drafted, the SLR by NOAA have been updated [39], including, among others: the elimination of the NOAA Extreme scenario due to better understanding of rapid ice sheet melt; a narrowing of the range between the other SLR scenarios because of increased certainty out to 2050; and a more than 10 fold increased frequency in coastal flooding projections over the next 30 years. For this manuscript, it means that the conclusions of this work, including outcomes of the 2017 workshops, will ultimately have to be re-examined and updated in light of the new science for the sustainability of life for humans and wildlife along with their environment and habitats. This approach also provides a more creative and simplistic means of communicating, sometimes difficult, climate science and modeled scenarios.

From a fish and wildlife and habitat perspective, impact assessments of climate change parameters need to be completed, using approaches as those presented here, for State and Federally listed, at risk, and indicator species to allow current information to be comprehensively incorporated into a suite of Federal and State wildlife conservation and action planning efforts. Additionally, continuous efforts to improve on stakeholder engagement, especially working with conservation partners, fish and wildlife scientists, local and regional planners, and climate experts, requires incorporation of “reflective practices” for the sustainability of these efforts. Reflections should focus on intentional examination of whether the goals, expectations, and sustainability of prior conservation and action planning efforts were met, what was learned (including insights gained and identifying changes to make in the future) and focus on identifying elements that are transferable to other conservation contexts. The world of climate change science is changing and advancing. As such, environmental managers should apply sustainable approaches such as the ones presented here to increase the soundness and better understanding of the science introduced into their decision-making processes.

### 4.2. Terrestrial Species—2017 Projections

Although a given SLR projection identifies a certain elevation for SLR at some time in the future, that elevation will be periodically reached before then because of short-term pulse events, such as king tides and storm surge [40]. Future climate change, especially SLR, effects to terrestrial plant and animal life, will be highly significant. The outcome of even the modest SLR scenarios indicates that salt water will begin to negatively affect the root zone of Big Pine Key’s last upland terrestrial vegetation between 2040 and 2060 with 0.6 m of SLR, and the island will be mostly underwater by 2060 to 2080 with 0.9 m of SLR. Vegetation succession will result in mangrove dominance on remaining land and development of more expansive estuaries across much of the island. After 2100, the lower elevation Keys will become all open-ocean habitat. These consequences of SLR in the long term will prove unsustainable and detrimental to the buttonwood and hardwood hammock habitats, important to many endemic species. Declines in capacity of freshwater storage and freshwater resources will be made worse with additive effects of storm surges and unusually high tides. these vegetative and urban transitions mean there is no transition space available for species and habitats, and thus unsustainable. As a result, most available adaptation actions call for creating temporary or permanent freshwater resources for terrestrial species, including the Florida Key deer [18]. After comparing the Benedict et al. [18] workshop findings with the NOAA 2017 [11] model scenarios and SLAMM modeling, the findings are very similar. The comparison among findings indicate that stressors can be exacerbated with short-term events like storm surge and tides. Another major area of agreement for sustainability is that multi-dimensional adaptation strategies will continue to be an ongoing process as sea levels rise, habitat changes, fresh water supplies diminish, and plants and animal populations decline.

### 4.3. Environmental Trends for Fish and Wildlife Management Consideratio—2017 Projections

Climatologic and resulting hydrologic changes will have huge effects on the ecology of the Florida Keys. The Florida Keys are going underwater with SLR, and surge events will exacerbate changes and challenges to ecosystem sustainability management. Even with near-term, interim adaptation strategies [18], beyond 0.9 m of SLR there are very few longer term sustainable options available since only 600 acres (243 ha) of Big Pine Key has elevations with undeveloped land (possible habitat) that would remain above water. The environmental trends identified from analyses in this study are relevant for consideration and potential incorporation into broader fish and wildlife and habitat analyses and planning. In addition to summarizing information related to model and scenario output for SLR, temperature, precipitation, tropical systems, and water demand estimations, a consideration of uncertainty is also relevant and important.

For this study, the 2017 NOAA Intermediate (0.3 m SLR), Intermediate-High (1.4 m SLR), and High (0.2 m SLR) SLR scenarios with projections out to 2100 were the most relevant scenarios for consideration. The Intermediate scenario is based on an analysis done by [41]. The Intermediate-High scenario is based on the latest assessment of NOAA 2017 [11] and the High scenario is based on loss of the Antarctic ice shelf predicted by NOAA 2017 [11]. For the Florida Keys, root zone salinization depths and timelines are an important analytical focus for SLR scenarios, rather than relying solely on surface inundation because SLR effects will occur decades earlier than surface inundation. Root zone inundation will vary by plant community but can often be at a depth of 0.25 m to 0.61 m. Big Pine Key’s last upland vegetation will be affected between 2040 and 2060. The island is projected to be underwater by 2060 to 2080.

For temperature consideration in habitat and species analyses and management actions, temperature increases in the State of Florida of 0.8 to 2.5 °C by 2050 and 1.7 to 3.9 °C by 2100 are expected. For precipitation consideration, higher fall and winter rainfall (dry season) by approximately 20%, and lower spring and summer rainfall (wet season) by approximately 30% is expected by 2100. According to Mgandu et al. [42], results from regression analyses show that rainfall is a significant predictor of water levels and climate change is responsible for 37% of changes in water levels.

The incorporation of urbanization changes into reviews or assessments, including both expected changes in population and land use/land cover, as well as characterization of sustainable changes in water demand is important for analyses. The role of water in human development is important, observed trends and climate forecasting provides evidence that water resources are vulnerable and will be strongly affected by climate change and industrialization which place key challenges to human existence [42]. For this study, the Water 2070 Technical Report [17] provided a multi-collaborator methodology for estimating sustainable water demand scenarios based upon several population change scenarios. Population-change scenarios provide a means of assessing the increasing fresh water demands and urbanization development impacts to habitat that can negatively impact terrestrial species. These urbanization impacts are worsened as SLR changes upland vegetation communities to more salt tolerant communities. At some point in the future, a tipping point will be reached with SLR causing a decrease in human population as developed and undeveloped dry land becomes inundated by salt water.

Consideration of scenario and human behavior uncertainties are important for evaluating the efficacy of climate projections. A common misconception is that the set of scenarios are based on one data set with varying degrees of uncertainty or probabilities. In fact, each graph line in a given scenario represents a different data set, highly dependent on the amount of greenhouse gas emissions that are currently being released, along with a projection of continued, reduced, or increased releases over time. As such, scenarios are intended to describe plausible conditions that support decision-making under human behavior uncertainty [43].

There is higher certainty in projections from 2050–2100 for SLR due to model consensus after the initial acceleration has been completely defined. The scenario “spread” at the timeline end of the scenarios (usually 2100) is not an indication of uncertainty or probabilities but rather a forecast of SLR due to varying greenhouse gas emission rates. There is a lower certainty from 2020–2050 due to global model uncertainty of acceleration rates, which began in the early 2000s. Specifically for this study, most scenarios for the time period of 2050–2100 predict a 0.9 to 1.8 m SLR, which would lead to the disappearance of most of the Florida Keys [40].

### 4.4. Future Needs

Future needs fall into four main categories: science needs related to regional climate projections; improving impact assessments for fish and wildlife and habitat resources; increased learning on continual improvement in stakeholder engagement; and advancing intentional conversations about adaptation management among scientists, resource managers, and other stakeholders. Examples of climate science needs include increase understanding of the newer science on melting of polar ice caps and its concomitant effects on SLR. The 2017-basedunderstanding is that even less than 2% ice melt will cause an additional 0.9 m of SLR [11]. At regional scales, improvements in visualizations of existing modeling tools for regional and local scales climate projections are needed to depict effects of the changing environment and SLR on connected socio-ecological systems. Future needs on improving impact assessments for fish and wildlife and habitat resources and needs on increased learning on continual improvement in stakeholder engagement are described in the Robustness of Approach section above. Finally, future analyses are needed to address adaptation recommendations for creating temporary or permanent freshwater resources for the Florida Key deer and other terrestrial species. The recent National Park Service guidance for climate-smart planning and management in the National Park Service [8] as well as discussions with local managers and stakeholders, is a good resource for adaptation management.

## Figures and Tables

**Table 1.** NOAA 2017 [11] scenarios with descriptions of when highest elevation root zone effects of SLR and SLR plus high tide (an additional 0.30 m and tidal flooding will occur in the Florida Keys. (from [28]).

Scenario	SLR (0.9 m)	SLR + Tide (0.9 m)	Earlier Inundation
Decreasing GHG–2060	2100	2085	+15 years earlier
Decreasing GHG–2080	2080	2065	+15 years earlier
Business as Usual	2065	2055	+10 years earlier
Potential Rapid Ice Melt	2060	2050	+10 years earlier

**Table 2.** SLAMM v. 6.4 model calculations of hectares and percent overall of: developed dry land (urbanization); available dry habitat (undeveloped upland vegetation and other dry land); and mangrove encroachment for the current baseline and SLR projections over time of 0.3 m, 0.6 m, 0.9 m, and 1.2 m of SLR [15] on Big Pine and No Name Keys.

	Current Baseline			0.3 m SLR (2030–2040)		
Land Cover	Hectatares (Current)	Percent (%) Remaining	Change %	Hectares (0.9 m SLR)	Percent (%) Remaining	Change %
Developed Dry Land	554 ha	15	-	495 ha	14	−10.6
Undeveloped Dry Land	1148 ha	32	-	935 ha	16	−18.5
Mangrove and Estuaries	563 ha	16	-	782 ha	22	+38.9
	0.6 m SLR (2050–2060)			0.9 m SLR (2060–2080)		
Land Cover	Hectares (0.9 m SLR)	Percent (%) Remaining	Change %	Hectares (0.9 m SLR)	Percent (%) Remaining	Change %
Developed Dry Land	359 ha	10	−35.2	164 ha	5	−70.3
Undeveloped Dry Land	559 ha	15	−51.3	243 ha	7	−78.8
Mangrove and Estuaries	1394 ha	39	+147.4	1562 ha	43	+177.2
	1.2 m SLR (2070–2090)					
Land Cover	Hectares (1.2 m SLR)	Percent (%) Remaining	Change %			
Developed Dry Land	71 ha	2	−87.2			
Undeveloped Dry Land	112 ha	3	−90.2			
Mangrove and Estuaries	1602 ha	44	+184.4			

## Data Availability

All results and secondary data mentioned in this article are publicly available and described in the References section.

## Appendix A. Summary of Climate Scenario Development

In 2017, the NOAA updated SLR models that are dependent on greenhouse gas (GHG) emission rates and other regional processes [11]. The 2017 NOAA Intermediate scenario was introduced while retaining 2017 NOAA Intermediate Low and 2017 NOAA Intermediate High. With increased SLR, in scenarios C3–C5 in particular, the 2017 NOAA Intermediate High scenario moved up and combined with the 2018 USACE High scenario. In 2017, the C1–C5 scenario nomenclature was no longer in use by the University of Florida GeoPlan SLR Sketch Tool [44]. The following are descriptions and relationships between the historical naming convention and updated scenarios for comparison [28]:

- C1—2017 NOAA Low Scenario–Decreasing GHG by 2020
- C2—2017 NOAA Intermediate Low/USACE Intermediate Scenario–Decreasing GHG by 2040
- C3—2017 NOAA Intermediate–Decreasing GHG by 2060
- C4—2017 NOAA Intermediate High/2018 USACE High–Decreasing GHG by 2080
- C5—2017 NOAA High–Business as Usual
- 2017 NOAA Extreme–Potential for Rapid Ice Melt

The SLR scenarios graphed in Figure 7 are based on IPCC 2013 GHG emission scenarios as regionally updated by NOAA 2017 [11]. The top line is the 2017 NOAA Extreme rate that represents accelerated polar ice sheet melt that has been recently documented [35]. The next red line is the 2017 NOAA High rate that represents “business as usual,” or GHG emissions of 2 ppm/year or greater. This means that GHG emission do not decrease over time. This line also incorporates some melting of the Antarctic ice shelf (95% confidence interval), which is new science within the NOAA 2017 [11] report. The orange line is the 2018 USACE High scenario and is also “business as usual” where GHG emissions continue to rise until 2080 (95% confidence interval) but without the input of any polar ice sheet melt. The polar ice sheet melt was unknown during the IPCC 2013 [13] scenario model runs. The crimson line is the 2017 NOAA Intermediate High rate that shows GHGs starting to decrease in 2060. The next two lines have current GHG emissions decreasing by 2040 and 2020, respectively (NOAA Intermediate Low and NOAA Low rates). The last line indicates a 2017 NOAA mathematical extrapolation from GHG emissions in 2000 with no other variables.

According to statistical analyses from [41,45–49] cited in the NOAA (2017) [11] report, the probability of exceeding the lowest scenario that considers an immediate reduction in GHG (0.3 m SLR by 2100) is 100%. The probability of exceeding the scenario of decreasing GHG in 2040 (0.49 m SLR by 2100) is 96%. Thus, these two scenarios, along with the 2000 extrapolation scenario, are eliminated from the current range of SLR scenarios in this analysis (Figure 7). Probability of the higher-end scenarios are not factored into the probability estimates included in NOAA (2017) [11] and are not yet available in the peer-reviewed literature. These high-end scenarios are contributions from ice-cliff and ice-shelf feedback processes that significantly increase ice-sheet contributions to GMSL rise, particularly under high emissions scenarios [50].

Newer information on scientifically supported SLR projections of IPCC (2013) [13] includes recent observational and modeling literature related to the potential for rapid ice melt in Greenland and Antarctica. The projections and results presented in several peer-reviewed publications provide evidence to support a GMSL rise in the range of 1.4 m to 2.7 m by 2100, and recent results regarding Antarctic ice-sheet instability indicate that such outcomes will be more likely than previously thought [11]. To ensure consistency with these recent updates to the peer-reviewed scientific literature, NOAA recommends a revised “extreme” upper-bound scenario for GMSL rise of 2.5 m by the year 2100, which is 0.46 m higher than the upper bound scenario from [43] and IPCC [13] employed by the NCA 2014 and 2018 [16,37]. The NOAA 2017 scenarios [11] for the time period of 2050–2100 predict a 0.9 to 1.8 m SLR with surface inundation in the Florida Keys [40].
